# Supplementary material for: An Investigation of the Neurotoxic Effects of Malathion, Chlorpyrifos, and Paraquat to Different Brain Regions
Source: Brain Sci. 2022 Jul 24;12(8):975. doi: 10.3390/brainsci12080975 (PMC9394375; doi:10.3390/brainsci12080975)
Supplement: Supplementary file 1 [file brainsci-12-00975-s001.zip › Supplementary Figure S1.pdf]

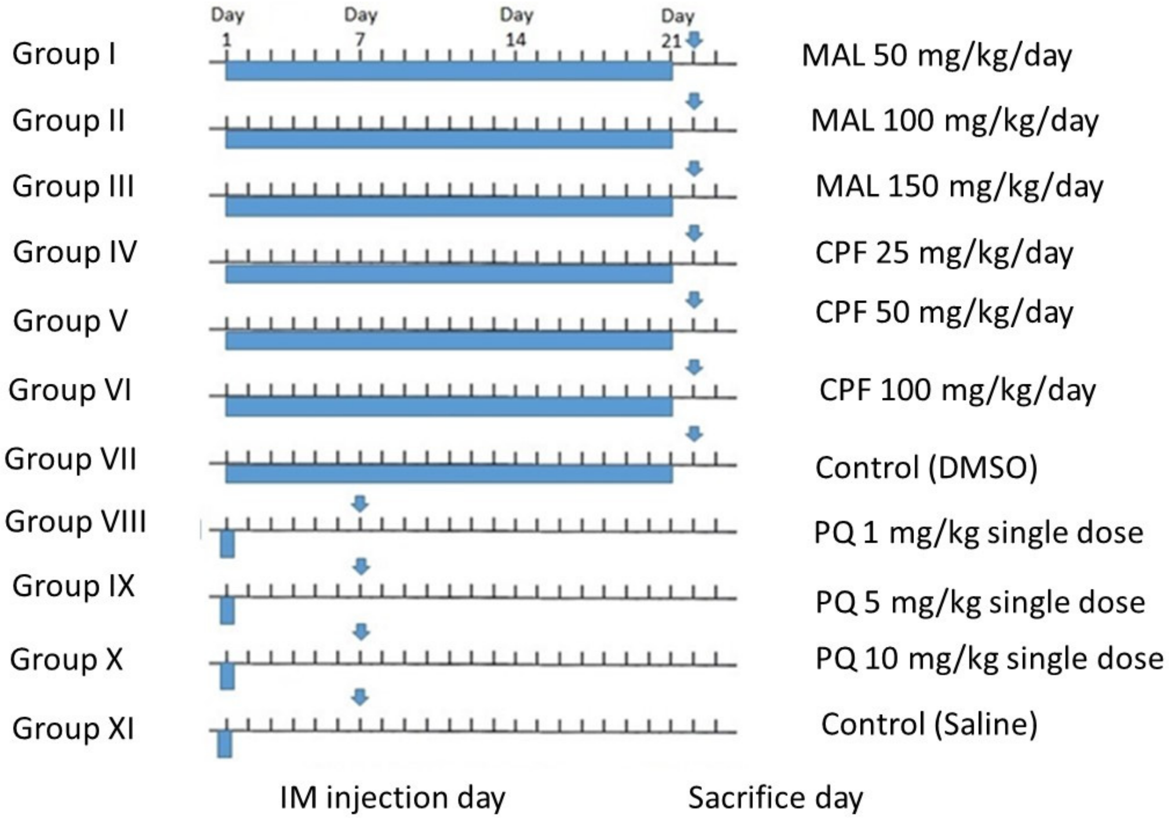

Supplementary Figure S1: Pesticide dosing regimens.

Sixty-six rats were used in this study, subdivided into 11 groups of 6 rats. Groups I to III received malathion (MAL) at a dose of either 50, 100, or 150 mg/kg, respectively. Groups IV to VI received chlorpyrifos at a dose of 25, 50, or 100 mg/kg, respectively. Group VII received DMSO as a vehicle control for the MAL and CPF treated groups. Groups VIII to X received PQ at a dose of 1, 5, or 10 mg/kg, respectively. Group XI received sterile saline as a vehicle control for the PQ treated groups. Pesticides, DMSO or saline were administered intramuscularly (IM) to the thigh quadriceps muscles each day for 21 days for the MAL, CPF, and DMSO (control) groups, while PQ (or saline control) were administered as a single dose. Twenty-four hours after the last dose of MAL and CPF, and 7 days after the single PQ injection, rats were sacrificed, and brain tissue removed for analysis.
