# Supplementary material for: An Investigation of the Neurotoxic Effects of Malathion, Chlorpyrifos, and Paraquat to Different Brain Regions
Source: Brain Sci. 2022 Jul 24;12(8):975. doi: 10.3390/brainsci12080975 (PMC9394375; doi:10.3390/brainsci12080975)
Supplement: Supplementary file 1 [file brainsci-12-00975-s001.zip › Supplementary Table S1.pdf]

Pesticide	Source of Variation	AChE<br>% of total variation (*p*-value)	NTE<br>% of total variation (*p*-value)
MAL	Interaction	6.73 (< 0.0001)	6.72 (< 0.0001)
	Dose	12.70 (< 0.0001)	41.26 (< 0.0001)
	Brain region	68.20 (< 0.0001)	20.48 (< 0.0001)
CPF	Interaction	6.90 (< 0.0001)	3.92 (0.0011)
	Dose	13.17 (< 0.0001)	40.68 (< 0.0001)
	Brain region	69.27 (< 0.0001)	18.28 (< 0.0001)
PQ	Interaction	7.17 (< 0.0001)	5.70 (< 0.0001)
	Dose	12.49 (< 0.0001)	48.55 (< 0.0001)
	Brain region	70.09 (< 0.0001)	15.51 (< 0.0001)

Supplementary Table S1: Association of pesticide-induced inhibition of acetylcholinesterase and neuropathy target esterase with pesticide dose and brain region.

Rats were dosed with malathion (MAL), chlorpyrifos (CPF), or paraquat (PQ) and the level of acetylcholinesterase (AChE) and neuropathy target esterase (NTE) activity quantified within the hippocampus, corpus striatum, cerebellum and cerebral cortex. A two-way ANOVA was performed to consider the association between pesticide inhibition of AChE and NTE, and pesticide dose and brain region.
