# Supplementary material for: An Investigation of the Neurotoxic Effects of Malathion, Chlorpyrifos, and Paraquat to Different Brain Regions
Source: Brain Sci. 2022 Jul 24;12(8):975. doi: 10.3390/brainsci12080975 (PMC9394375; doi:10.3390/brainsci12080975)
Supplement: Supplementary file 1 [file brainsci-12-00975-s001.zip › Supplementary Table S2.pdf]

		Control	MAL 50 mg/kg	MAL 100 mg/kg	MAL 150 mg/kg	CPF 25 mg/kg	CPF 50 mg/kg	CPF 100 mg/kg	PQ 1 mg/kg	PQ 5 mg/kg	PQ 10 mg/kg
Mitochondrial complex I		Hippocampus									
	Vmax	0.48 ± 0.03	0.43 ± 0.01	0.38 ± 0.03	0.65 ± 0.06	0.43 ± 0.01	0.38 ± 0.03	0.65 ± 0.06	0.48 ± 0.02	0.44 ± 0.00	0.45 ± 0.03
	Ki	37.9 ± 5.5	56.72 ± 3.4	70.19 ± 11.4	309.40 ± 39.3	56.7 ± 3.4	70.2 ± 11.39	309.40 ± 39.3	44.7 ± 4.4	65.2 ± 0.9	117.1 ± 14.04
		Corpus striatum									
	Vmax	0.43 ± 0.03	0.43 ± 0.03	0.38 ± 0.03	0.39 ± 0.04	0.43 ± 0.03	0.38 ± 0.03	0.39 ± 0.04	0.41 ± 0.02	0.39 ± 0.01	0.39 ± 0.02
	Ki	30.79 ± 6.1	56.71 ± 7.9	76.19 ± 13.6	141.90 ± 22.9	56.71 ± 7.9	76.19 ± 13.6	141.90 ± 22.9	37.07 ± 4.5	59.96 ± 3.1	117.60 ± 11.2
		Cerebellum									
	Vmax	0.46 ± 0.02	0.40 ± 0.01	0.42 ± 0.02	0.44 ± 0.03	0.36 ± 0.01	0.40 ± 0.02	0.34 ± 0.03	0.45 ± 0.01	0.42 ± 0.00	0.42 ± 0.02
	Ki	35.98 ± 4.2	39.98 ± 3.9	64.11 ± 5.30	104.90 ± 15.5	47.27 ± 2.6	104.50 ± 9.8	153.10 ± 22.0	41.81 ± 3.3	59.93 ± 1.4	99.98 ± 7.55
		Cerebral cortex									
	Vmax	0.52 ± 0.02	0.50 ± 0.01	0.43 ± 0.01	0.51 ± 0.05	0.51 ± 0.01	0.40 ± 0.03	0.34 ± 0.05	0.50 ± 0.02	0.47 ± 0.01	0.46 ± 0.03
	Ki	38.8 ± 4.62	59.1 ± 2.80	70.58 ± 4.28	160.40 ± 24.3	74.4 ± 3.01	88.3 ± 15.1	149.8 ± 36.9	43.5 ± 4.60	63.67 ± 2.1	107.7 ± 11.7
Mitochondrial complex III		Hippocampus									
	Vmax	0.49 ± 0.01	0.47 ± 0.01	0.43 ± 0.02	0.44 ± 0.05	0.46 ± 0.02	0.48 ± 0.02	0.57 ± 0.03	0.48 ± 0.01	0.43 ± 0.02	0.38 ± 0.05
	Ki	29.71 ± 1.94	34.92 ± 2.53	43.63 ± 4.95	72.74 ± 18.88	41.70 ± 4.15	61.87 ± 6.59	155.30 ± 14.47	33.78 ± 1.30	43.63 ± 4.95	61.17 ± 16.90
		Cerebral cortex									
	Vmax	0.45 ± 0.45	0.39 ± 0.01	0.36 ± 0.02	0.44 ± 0.07	0.39 ± 0.01	0.36 ± 0.02	0.44 ± 0.07	0.41 ± 0.41	0.38 ± 0.38	0.35 ± 0.35
	Ki	24.27 ± 1.27	36.01 ± 1.45	51.06 ± 5.15	141.8 ± 8.93	36.01 ± 1.45	51.06 ± 5.15	141.80 ± 12.93	29.33 ± 2.33	42.61 ± 2.61	72.95 ± 2.95
		Cerebellum									
	Vmax	0.45 ± 0.01	0.44 ± 0.01	0.41 ± 0.02	0.44 ± 0.01	0.44 ± 0.01	0.41 ± 0.02	0.44 ± 0.01	0.40 ± 0.01	0.47 ± 0.03	0.41 ± 0.01

	Ki	20.98 ± 1.62	30.56 ± 2.20	41.78 ± 4.29	78.43 ± 5.12	30.56 ± 2.20	41.78 ± 4.29	78.43 ± 5.12	25.93 ± 2.46	52.20 ± 8.58	72.86 ± 5.52
		Corpus striatum									
	Vmax	0.53 ± 0.02	0.50 ± 0.01	0.49 ± 0.02	0.56 ± 0.02	0.50 ± 0.01	0.49 ± 0.02	0.56 ± 0.02	0.52 ± 0.01	0.49 ± 0.02	0.44 ± 0.04
	Ki	31.02 ± 2.89	42.84 ± 2.18	62.80 ± 5.52	129.30 ± 9.96	42.84 ± 2.18	62.80 ± 5.52	129.30 ± 9.96	35.11 ± 2.43	47.28 ± 3.91	63.83 ± 12.44

Supplementary Table S2: Pesticide effects on mitochondrial complex I and complex III activites.

Rats were dosed with malathion (MAL), chlorpyrifos (CPF), or paraquat (PQ) and the activities of mitochondrial complex I and complex III quantified. A Michaelis-Menten model was used to determine values for the enzymatic maximal velocity (Vmax) and inhibitor constant (Ki) at each of the dosing levels and within the hippocampus (HC), corpus striatum (CS), cerebellum (CER) and cerebral cortex (CC).
